# A Replicating Cytomegalovirus-Based Vaccine Encoding a Single Ebola Virus Nucleoprotein CTL Epitope Confers Protection against Ebola Virus

**DOI:** 10.1371/journal.pntd.0001275

**Published:** 2011-08-09

**Authors:** Yoshimi Tsuda, Patrizia Caposio, Christopher J. Parkins, Sara Botto, Ilhem Messaoudi, Luka Cicin-Sain, Heinz Feldmann, Michael A. Jarvis

**Affiliations:** 1 Laboratory of Virology, Division of Intramural Research, National Institute of Allergy and Infectious Diseases, National Institutes of Health, Hamilton, Montana, United States of America; 2 Vaccine and Gene Therapy Institute, Oregon Health and Science University, Portland, Oregon, United States of America; 3 Department of Vaccinology and Applied Microbiology, Helmholtz Centre for Infection Research, Braunschweig, Germany; 4 Department of Molecular Microbiology and Immunology, Oregon Health and Science University, Portland, Oregon, United States of America; University of Texas Medical Branch at Galveston, United States of America

## Abstract

**Background:**

Human outbreaks of Ebola virus (EBOV) are a serious human health concern in Central Africa. Great apes (gorillas/chimpanzees) are an important source of EBOV transmission to humans due to increased hunting of wildlife including the ‘bush-meat’ trade. Cytomegalovirus (CMV) is an highly immunogenic virus that has shown recent utility as a vaccine platform. CMV-based vaccines also have the unique potential to re-infect and disseminate through target populations regardless of prior CMV immunity, which may be ideal for achieving high vaccine coverage in inaccessible populations such as great apes.

**Methodology/Principal Findings:**

We hypothesize that a vaccine strategy using CMV-based vectors expressing EBOV antigens may be ideally suited for use in inaccessible wildlife populations. To establish a ‘proof-of-concept’ for CMV-based vaccines against EBOV, we constructed a mouse CMV (MCMV) vector expressing a CD8^+^ T cell epitope from the nucleoprotein (NP) of *Zaire ebolavirus* (ZEBOV) (MCMV/ZEBOV-NP_CTL_). MCMV/ZEBOV-NP_CTL_ induced high levels of long-lasting (>8 months) CD8^+^ T cells against ZEBOV NP in mice. Importantly, all vaccinated animals were protected against lethal ZEBOV challenge. Low levels of anti-ZEBOV antibodies were only sporadically detected in vaccinated animals prior to ZEBOV challenge suggesting a role, at least in part, for T cells in protection.

**Conclusions/Significance:**

This study demonstrates the ability of a CMV-based vaccine approach to protect against an highly virulent human pathogen, and supports the potential for ‘disseminating’ CMV-based EBOV vaccines to prevent EBOV transmission in wildlife populations.

## Author Summary

Human outbreaks of hemorrhagic disease caused by Ebola virus (EBOV) are a serious health concern in Central Africa. Great apes (gorillas/chimpanzees) are an important source of EBOV transmission to humans. Candidate EBOV vaccines do not spread from the initial vaccinee. In addition to being highly immunogenic, vaccines based on the cytomegalovirus (CMV) platform have the unique potential to re-infect and disseminate through target populations. To explore the utility of CMV-based vaccines against EBOV, we constructed a mouse CMV (MCMV) vector expressing a region of nucleoprotein (NP) of *Zaire ebolavirus* (ZEBOV) (MCMV/ZEBOV-NP_CTL_). MCMV/ZEBOV-NP_CTL_ induced high levels of long-lasting CD8^+^ T cells against ZEBOV NP in mice. Importantly, all vaccinated animals were protected against lethal ZEBOV challenge. The absence of ZEBOV neutralizing and only low, sporadic levels of total anti-ZEBOV IgG antibodies in protected animals prior to ZEBOV challenge indicate a role, albeit perhaps not exclusive, for CD8^+^ T cells in mediating protection. This study demonstrates the ability of a CMV-based vaccine approach to protect against ZEBOV, and provides a ‘proof-of-concept’ for the potential for a ‘disseminating’ CMV-based EBOV vaccine to prevent EBOV transmission in wild animal populations.

## Introduction

Ebola virus (EBOV), a member of the *Filoviridae* family, causes rapidly progressing viral hemorrhagic fever culminating in multi-organ failure, shock and death [1]. EBOV can be subdivided into four distinct and a fifth putative species [2], [3]. EBOV species differ in level of virulence, with *Zaire ebolavirus* (ZEBOV) being the most virulent (80–90% case fatality) [4]. The unpredictable nature of EBOV outbreaks in endemic areas of Africa, combined with the potential for accidental and deliberate introduction into non-endemic nations ensures that EBOV will most likely remain a global health concern well into the future. Potential for rapid dissemination to non-endemic countries was demonstrated in 2008 by importation of Marburg virus (a filovirus closely related to EBOV) to the US [5] and Netherlands [6] by tourists infected in Uganda.

Animal species involved in EBOV transmission to humans are not completely defined [7]. Asymptomatically infected fruit bats have been identified during EBOV outbreaks, suggesting that bats may be a reservoir [8]. EBOV infection is also observed in great apes (chimpanzees/gorillas), where it is highly pathogenic with a similar disease course to humans [9]–[11]. Handling and butchering of EBOV-infected wildlife and carcasses including great apes is an important mode of transmission to humans [7], [9], [12], [13]. In the eighteen outbreaks of EBOV in Africa since its discovery in 1976, three were associated with exposure to environments inhabited by bats, and seven resulted from contact with great ape carcasses (the source of the remaining outbreaks was not established) [3], [7]. Although EBOV was identified in fruit bats in the EBOV outbreaks of 2001 and 2003, all known transmissions to humans resulted from handling great ape carcasses [9]. Due to its high pathogenicity in great apes, EBOV infection is also regarded as a major threat to the survival of great ape species in the wild [9]–[11].

Given the threat by EBOV for the extinction of great apes and the role of great apes in EBOV transmission to humans, vaccination of these animals in the wild has been proposed to save these endangered wildlife species and to reduce the incidence of human EBOV outbreaks [7], [14]. A number of candidate EBOV vaccines have been developed that are protective against infection in animal models [15], [16]. Replication-defective adenovirus (Ad) expressing EBOV glycoprotein (GP) alone [17] or in combination with nucleoprotein (NP) [18], virus-like particles comprised of virus matrix protein (VP40) and GP with or without NP [19], [20], and replication-competent human parainfluenza virus type 3 (HPIV3) [21], and vesicular stomatitis virus (VSV) expressing GP [22], [23] are all able to consistently induce protective immunity in small animal and non-human primate (NHP) models. Oral immunization with the VSV-based vaccine has been shown to induce protection in mice and NHPs [24], [25], leading to the suggestion of its use for food baiting [7], [14]. However, all of these EBOV vaccine approaches induce immunity only in the vaccinated individual as they are unable to disseminate through the population.

A ‘disseminating’ cytomegalovirus (CMV)-based vaccine offers an alternative approach whereby high coverage would be achieved by vaccine spread from initial vaccinees through the target population by animal-to-animal contact. CMV is an ubiquitous, but benign β-herpesvirus that establishes life-long, latent/low level persistent infection within the host [26]. During latent infection the virus is believed to be maintained as an extrachromosomal chromatin-complexed episome [27]. After initial infection, CMV is shed from epithelial surfaces into body fluids (saliva, urine, genital secretions and breast milk), and transmission generally involves mucosal exposure to such fluids, most commonly in early childhood or adolescence [28], [29]. CMV possesses the remarkable ability to reinfect and establish a persistent infection regardless of host CMV immunity [30]–[33]. CMV is also one of the most immunogenic viruses known [34], inducing a characteristic immune response that is highly enriched for ‘effector’ memory (T_EM_) T cells [33]. T_EM_ cell localization is shifted toward non-lymphoid, mucosal sites, and T_EM_ cells are functionally primed for immediate anti-pathogen effector function [35]. Due to this high immunogenicity, interest in developing CMV as a vaccine vector is increasing [36]–[38]. The recent capacity to manipulate the CMV genome using bacterial artificial chromosome (BAC)-based technology has facilitated development of CMV as a vaccine vector [39]. To date, target antigens have been expressed in CMV either as single T cell epitopes fused to a non-essential CMV gene [36], or as single full-length proteins under the control of heterologous promoters [33], [40]. CMVs are host-specific, with each mammalian host being infected with its own distinct CMV [41], [42]. The high efficacy of CMV-based vaccines was recently demonstrated by the ability of a panel of rhesus CMV (RhCMV)-based vectors each expressing a distinct simian immunodeficiency virus (SIV) antigen to prevent systemic SIV infection of rhesus macaques (a NHP model for HIV), which is the first vaccine to prevent acquisition of fully pathogenic SIV [33], [43].

Our long-term goal is to develop a ‘disseminating’ vaccine against EBOV based on chimpanzee/gorilla-specific CMV vectors that will prevent EBOV infection in gorillas and chimpanzees. We hypothesize that protection of these animals from EBOV will interfere with the transmission of EBOV from these species to humans. In the present report we have constructed a mouse CMV (MCMV)-based EBOV vector expressing a single CTL epitope from NP of ZEBOV (MCMV/ZEBOV-NP_CTL_) as a prototype vector to establish ‘proof-of-concept’ for this approach. MCMV/ZEBOV-NP_CTL_ was shown to be highly immunogenic, inducing durable CD8^+^ CTL responses (IFNγ^+^/TNFα^+^) against ZEBOV NP in multiple strains of mice. Importantly, MCMV/ZEBOV-NP_CTL_ conferred protection against lethal challenge with a mouse-adapted ZEBOV variant. The general absence of antibodies against ZEBOV in protected animals prior to ZEBOV challenge, and lack of protection in controls receiving wild-type (WT) ‘empty’ MCMV vector were consistent with protection being, at least in part, T cell-mediated. This is the first study to demonstrate the ability of a CMV-based vaccine to protect against an human pathogen, and supports the concept of ‘disseminating’ CMV-based EBOV vaccines to prevent EBOV transmission in wild animal populations.

## Materials and Methods

### Ethics statement

All animal use complied with the Guide for the Use and Care of Laboratory Animals, USDA Animal Welfare Regulations, PHS Policy on Humane Care and Use of Laboratory Animals and other relevant regulations. All procedures were approved by the respective IACUC committees at Rocky Mountain Laboratories, Division of Intramural Research, National Institute of Allergy and Infectious Diseases, National Institutes of Health (RML, DIR, NIAID, NIH), and Oregon Health and Science University (OHSU).

### Construction and characterization of MCMV vectors

MCMV-based vectors were constructed by lambda-phage based linear recombination using a strategy identical to that used for construction of other CMV recombinants [33]. MCMV (Smith strain) BAC pSMfr3 [44], [45] in which the natural killer (NK) cell activating m157 MCMV gene has been deleted (pSMfr3Δm157) was used as the genetic background for these vectors. Deletion of m157 was necessary to avoid attenuation of CMV replication by inadvertent high NK cell control in the C57BL/6 mouse strain that expresses the corresponding Ly49H NK receptor [46]. For construction of MCMV/ZEBOV-NP_CTL_ an H2^b^-restricted T cell epitope from NP of ZEBOV (43-VYQVNNLEEIC-54) [47], [48] was fused ‘in-frame’ to the carboxyl terminus of MCMV IE2 (*ie2*) generating the recombinant MCMV BAC, pMCMV/ZEBOV-NP_CTL_. IE2 is a nonessential MCMV protein to which we and others have fused defined T cell epitopes as a strategy for induction of T cell responses following infection of mice with the corresponding recombinant MCMV [36]. A contiguous *frt*-flanked kanamycin resistance marker (Kan^R^) was inserted into the MCMV BAC genome at the same time as the NP epitope to enable selection of recombinant BACs on the basis of kanamycin resistance. Following selection of recombinant BACs on the basis of Kan^R^, the *frt*-flanked Kan^R^ marker was removed by arabinose induction of Flp-recombinase and screening for kanamycin sensitivity. Virus was reconstituted from BACs by transfection into murine embryo fibroblasts (MEFs). Presence of the BAC cassette within the MCMV genome decreases *in vivo* replication, and serial *in vitro* passage of the BAC-derived virus was performed to remove the BAC cassette [45]. Absence of the BAC cassette from reconstituted MCMV vectors was confirmed by PCR using BAC cassette-specific primers. MCMV/ZEBOV-NP_CTL_ viruses were assessed for growth *in vitro* on MEFs. To avoid effects of inadvertent second site mutations, two independently derived MCMV/ZEBOV-NP_CTL_ clones (5A1 and 5D1) were selected, and growth was compared to WT MCMV (MCMVΔm157). For assessment of virus growth kinetics, cells were infected at a multiplicity of infection (MOI) of 0.1 and media was harvested for quantitation of virus at increasing times post-infection by standard plaque assay. DNA sequencing of BAC and reconstituted viral DNA was used to confirm integrity of the NP epitope within the MCMV genome.

### Animal models, vaccination and challenge

Mice were purchased from NCI at Frederick, MD. All experiments were performed with age-matched female 129S1/SvlmJ/Cr and C57BL/6 mice. Mice were provided food and water *ad libitum*. For analysis of the kinetics of the peripheral blood anti-NP T cell response, mice received a single intraperitoneal (i.p.) inoculum of MCMV/ZEBOV-NP_CTL_ (1×10^5^ pfu), and were then bled over a 33 week period at times indicated. In all other cases, MCMV-vaccinated mice were inoculated i.p. with 5×10^5^ pfu of MCMV recombinants followed by an identical i.p. ‘boost’ after 4 weeks. After 10 weeks, C57BL/6 mice were challenged i.p. with 10^3^ LD_50_ of mouse-adapted ZEBOV (ma-ZEBOV) as previously described [24]. For these studies, VSVΔG/ZEBOVGP (given as a single i.p. dose of 5×10^5^ pfu) served as a positive control for vaccine protection. The VSVΔG/ZEBOVGP is a recombinant VSV, in which the native glycoprotein G has been exchanged for GP ZEBOV (Mayinga strain), and has been shown to induce protective immunity against ma-ZEBOV [24]. Following challenge, disease severity was monitored on the basis of clinical signs using an approved scoring index, and mortality rate was recorded over the 28 day post-challenge period. ZEBOV *in vivo* replication was also directly determined by quantification of ma-ZEBOV viremia levels at time of peak viremia in mice (day 4 post-challenge) by virus titration using a focus forming unit immunodetection assay with titre expressed as focus forming units (FFU/ml). All BSL-4 level infectious work was performed at RML.

### Intracellular cytokine staining analysis of T cells

Frequencies of CD8^+^ T cells directed against the ZEBOV NP CTL epitope, or MCMV-encoded M38 and M45 proteins in pooled peripheral blood or spleen were determined by intracellular cytokine staining (ICS). Cells were stimulated in the presence of brefeldin A (BFA) (10 µg/ml) with peptides representing defined H2^b^-restricted epitopes of ZEBOV NP (VYQVNNLEEIC) [47], MCMV M38 (SSPPMFRV) or M45 (HGIRNASFI) [49], or prostate-specific antigen (PSA) (HCIRNKSVI) [50]. PSA was used as an irrelevant antigen control, and the peptides representing the well-characterized H2^b^-restricted epitopes in MCMV-encoded M45 and/or M38 [49] served as an indicator of MCMV vector infection. Incubation without antigen served as a background control. After 6 hours of stimulation, cells were stained using the following monoclonal antibodies (Mabs) in designated combinations: a) from BD Biosciences, RM4-5 (CD4; Pacific Blue), 53-6.7 (CD8a; PerCP-Cy5.5), MP6-XT22 (TNFα; PE), XMG1.2 (IFNγ; APC), and b) from eBioscience 17A2 (CD3e, APC-eFluor780). After surface and intracellular staining with conjugated Mabs, polychromatic flow cytometric analysis was performed on a LSR II (BD Biosciences), and data was analyzed by using FlowJo software (version 9.1; Tree Star, Inc.). Samples were performed in triplicate. Response frequencies were determined by subtracting background and then averaging background subtracted responses.

### Neutralization assay

Sera was collected from vaccinated mice at times indicated, and analyzed for ability to neutralize ZEBOV infection in an *in vitro* neutralization assay [51]. Briefly, heat inactivated sera (56°C for 45 minutes) was serially diluted in DMEM, and then mixed 1∶1 with ZEBOV expressing the EGFP reporter (ZEBOV-EGFP) (200 FFU/well). After incubation at 37°C for 60 minutes, 20 µl of the mixture was transferred onto subconfluent Vero cells in a 96-well plate format and incubated for 30 minute at 37°C. Following addition of 180 µl of DMEM supplemented with 1.5% carboxymethyl cellulose and 5% FBS, cells were cultured for 4 days at 37°C. Cells were washed by PBS and fixed in 10% neutral buffered formalin overnight under BSL-4 conditions. Prior to removal from the BSL-4 conditions, formalin was changed and plates were processed under BSL-2 conditions by conventional methods. Values shown are the sera dilutions resulting in 50% reduction in EGFP-positive cells following infection of Vero cells with ZEBOV-EGFP. Mab#226 is a neutralizing mouse monoclonal antibody made against ZEBOV GP [52].

### Virus-like particle (VLP)-based enzyme-linked immunosorbent assay (ELISA)

Total IgG antibody responses to ZEBOV NP, GP and VP40 were quantified by ELISA using ZEBOV VLPs (VP40/NP/GP) as an antigen source [25]. Generation of VLPs has been previously described [53]. Briefly, 96 well microtiter plates (NUNC, Rochester, NY) were coated with ZEBOV VLPs (2 µg/ml) in PBS at 4°C overnight, and then blocked with 5% skim milk in PBS containing 0.05% Tween 20 (PBST) for 2 hours at room temperature. After three washes with PBST, 50 µl of diluted heat-inactivated serum sample was added and the plates were incubated for 1 hour at 37°C. After an additional three washes with PBST, secondary antibody conjugated with horseradish peroxidase (HRP) was added and plates were incubated for an additional 1 hour at 37°C. Bound antibodies were quantified using the ABTS Peroxidase Substrate System (KPL, Gaithersburg, MD) by measuring absorbance at 405 nm on a microplate spectrophotometer. Values shown are the end-point dilution titre (using a 4-fold dilution series). Samples were deemed positive when the value was higher than the mean plus 4 standard deviations of negative (Mock) mouse sera [25].

### Statistical analysis

Statistical analyses were performed using GraphPad version 5.0 d for Mac OS X, GraphPad Software, San Diego CA, USA, www.graphpad.com. An unpaired Student's two-tailed t-test was used to compare treatment groups. A Kaplan-Meier estimator and a log-rank test were used to compare survival rates between treatment groups in ma-ZEBOV challenge studies.

## Results and Discussion

**Figure 1:**
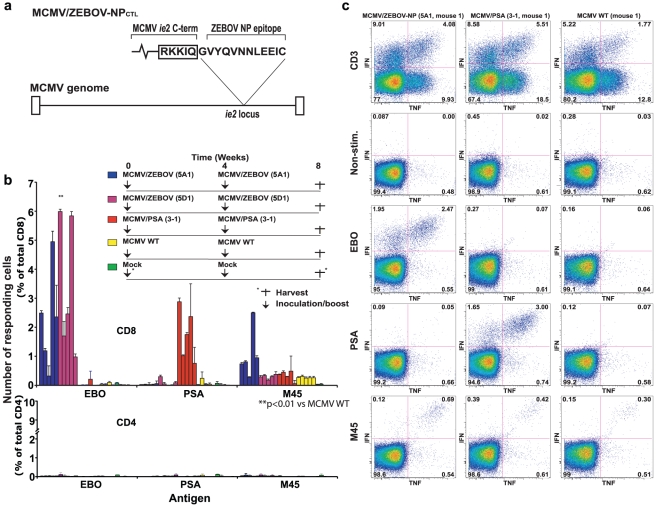
T cell responses following immunization with MCMV/ZEBOV-NP_CTL_. (a) Schematic representation of MCMV/ZEBOV-NP_CTL_. An H2^b^-restricted T cell epitope from ZEBOV NP (VYQVNNLEEIC) was fused ‘in-frame’ to the carboxyl terminus of MCMV IE2 (*ie2*) generating the recombinant MCMV, MCMV/ZEBOV-NP_CTL_. MCMV IE2 is a non-essential protein. (b) 129S1/SvlmJ/Cr (H2^b^-restricted) mice (n = 5/group) were immunized i.p. using 5×10^5^ pfu of the following: one of two independent clones of MCMV/ZEBOV-NP_CTL_ (5A1 and 5D1), MCMV/PSA (clone 3-1) (a comparable MCMV vector expressing IE2 fused to an H2^b^-restricted epitope from PSA), WT MCMV, or diluent (Mock control). Mice were boosted after 4 weeks. After 8 weeks splenocytes were harvested for analysis of T cell responses. T cells were analyzed by using ICS with a 6 hour incubation in the presence of BFA with peptide (or anti-CD3 Mab, for total T cell response). Levels of responding (IFNγ and TNFα double-positives) CD8^+^ (top) and CD4^+^ (bottom) cells in individual mice are shown. All MCMV/ZEBOV-NP_CTL_ immunized mice (n = 10) showed significant CD8-restricted T cell responses against the NP target antigen. (c) Typical responses from MCMV/ZEBOV-NP_CTL_ vaccinated mice. The majority of ZEBOV NP-responding T cells expressed both IFNγ and TNFα and are specific for the NP epitope (not observed following incubation with the PSA peptide or unstimulated controls). Consistent with MCMV infection, all mice demonstrate T cell responses to MCMV M45. T cell responses directed against M45 are known to be ‘non-inflationary’, generally representing <1% of total CD8^+^ T cells during chronic MCMV infection. Error bars show the standard deviation (s.d.).

To assess the potential of CMV for development as a vaccine against EBOV, we designed a prototype murine cytomegalovirus (MCMV)-based EBOV vaccine (MCMV/ZEBOV-NP_CTL_) expressing a CD8^+^ CTL epitope from ZEBOV NP (43-VYQVNNLEEIC-53; NP_43_) [47], [48], [54] fused to a non-essential MCMV protein, IE2 (Figure 1a). MCMV/ZEBOV-NP_CTL_ was constructed by lambda-based linear recombination using a BAC containing the MCMV genome (pSM3fr) [33], [45]. Independent pMCMV/ZEBOV-NP_CTL_ clones (5A1 and 5D1) were selected for characterization. Restriction enzyme digestion followed by electrophoresis showed no gross genomic rearrangements compared to WT parental BAC (Figure S1). Viruses were reconstituted by transfection of BAC DNA into MEFs. *In vitro* growth analysis of reconstituted viruses showed replication kinetics comparable to WT MCMV (Figure S2).

CMV induces high levels of T cells against both endogenous and heterologously expressed proteins [33], [34], [36]. To assess the level of NP-specific CD8^+^ CTL responses induced by MCMV/ZEBOV-NP_CTL_, we performed immunogenicity studies in H2^b^-restricted 129S1/SvlmJ/Cr mice. Mice (n = 5/group) were immunized intraperitoneally (5×10^5^ pfu; i.p.) with MCMV/ZEBOV-NP_CTL_ (clone 5A1 or 5D1), MCMV/PSA (clone 3-1), WT MCMV or diluent (Mock). MCMV/PSA (clone 3-1) is a control MCMV expressing an irrelevant H2^b^-restricted epitope from PSA [50]. After 4 weeks, mice were ‘boosted’ using an identical inoculum. After 8 weeks, splenocytes were harvested for analysis of T cell responses (Figure 1b). Antigen-specific T cells were analyzed by ICS following a 6 hour *in vitro* incubation with EBOV and MCMV peptides representing different H2^b^-restricted epitopes. All MCMV/ZEBOV-NP_CTL_ vaccinated mice exhibited significant CD8^+^ CTL responses against ZEBOV NP (Figure 1b). The level of NP responses elicited by 5A1 and 5D1 were not significantly different, and were considered together as a single data set. The ZEBOV NP-specific T cell responses induced were substantial (mean = 2.83% of total CD8^+^ T cells; range = 0.32 to 5.99%), CD8^+^ phenotype (no response in CD4^+^ cell compartment), and specific (directed against ZEBOV NP, but not PSA control). CD8^+^ CTLs induced against ZEBOV NP primarily expressed both IFNγ and TNFα effector cytokines (Figure 1c). All mice except mock-vaccinated controls had CD8^+^ CTLs directed against the MCMV-encoded M45 protein.

**Figure 2:**
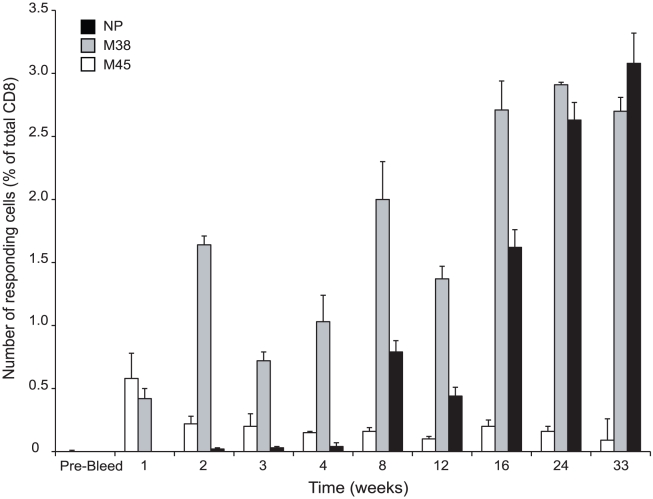
Kinetic analysis of CD8^+^ T cell response to MCMV/ZEBOV-NP_CTL_. 129S1/SvlmJ/Cr H2^b^-restricted mice (n = 14) were immunized (i.p.) with a single dose (1×10^5^ pfu) of MCMV/ZEBOV-NP_CTL_ (clone 5D1). At times indicated, mice were bled and peripheral T cell responses were measured in pooled blood by using ICS with a 6 hour incubation in the presence of BFA with peptides. All responses were normalized against cells stimulated in the absence of peptide. Responses are against ZEBOV NP (black), or MCMV M38 (grey) and M45 (white). Error bars show the s.d.

A unique characteristic of CMV-induced immune responses is their ‘inflation’ over time with maturation into stable ‘effector’ T cell (T_EM_) memory that persists for life [55]. Compared to classical ‘central’ memory (T_CM_) cells, T_EM_ are biased toward localization at mucosal epithelial effector sites, and have more immediate effector function [56], [57]. To determine the durability of ZEBOV NP-specific T cell responses from a single MCMV/ZEBOV-NP_CTL_ inoculation, mice (n = 14) were vaccinated (1×10^5^ pfu; i.p.) with MCMV/ZEBOV-NP_CTL_, and peripheral T cell responses were followed longitudinally. NP-specific CD8^+^ T cell responses gradually accumulated to high levels and persisted (increasing from 0.79% after 8 weeks, to 3.08% after 33 weeks following the single inoculation) (Figure 2). Although delayed, the NP-specific CTL response was comparable in kinetics of induction and magnitude to the T_EM_-biased ‘inflationary’ response directed against MCMV M38 [49], [58]. Importantly, these results show that a CMV-based EBOV vaccine can induce high levels of CD8^+^ T cells against an EBOV antigen that increase with time and are durable.

**Figure 3:**
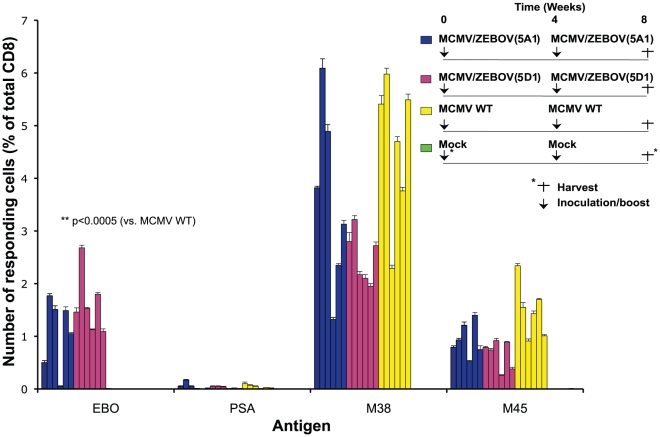
MCMV/ZEBOV-NP_CTL_ induces a ZEBOV-specific T cell response in C57BL/6 mice. C57BL/6 H2^b^-restricted mice (n = 6/group) were vaccinated (i.p.) using 5×10^5^ pfu of MCMV/ZEBOV-NP_CTL_ clone 5A1 or 5D1. Control groups received either MCMV WT, or diluent (Mock). After 4 weeks mice were boosted as before. After 8 weeks (4 weeks post-boost) mice were harvested for analysis of splenocyte T cell responses by ICS using a 6 hour incubation in the presence of BFA with indicated peptide. MCMV-specific CD8^+^ T-cell responses against MCMV M45 and M38 were used as markers of MCMV infection. PSA peptide served as an H2^b^-restricted epitope specificity control. Responding CD8^+^ cells shown are IFNγ and TNFα double-positives. Mice groups presented in this figure were vaccinated in parallel with mice groups (n = 14/group) used to ascertain protective efficacy of vaccination regimen shown in Figure 4. Error bars show the s.d.

To determine whether MCMV/ZEBOV-NP_CTL_ was able to induce protective immunity against lethal ZEBOV challenge, we performed challenge studies in C57BL/6 mice using ma-ZEBOV [24], [59]. The ma-ZEBOV is lethal in unvaccinated mice, which succumb 5–7 days post-challenge [24], [59]. Four groups of mice (n = 20/group) were immunized (5×10^5^ pfu; i.p.) with MCMV/ZEBOV-NP_CTL_ 5A1 or 5D1, MCMV WT or diluent, and boosted after 4 weeks (Figure 3). After 8 weeks (4 weeks following the boost), splenocytes from 6 mice/group were analyzed for T cell responses. 5A1 and 5D1 induced comparable responses against NP, enabling mice receiving either clone to be considered as a single data set. MCMV/ZEBOV-NP_CTL_ induced considerable levels of CD8^+^ T cells against ZEBOV NP (mean = 1.34% of total CD8^+^ T cells; range = 0.05 to 2.68%). These results also show that the ability of MCMV/ZEBOV-NP_CTL_ to induce NP-specific T cells is independent of the mouse strain (Figures 1b and 3). All mice except mock-vaccinated controls had CD8^+^ CTLs directed against MCMV-encoded M38 and M45.

**Figure 4:**
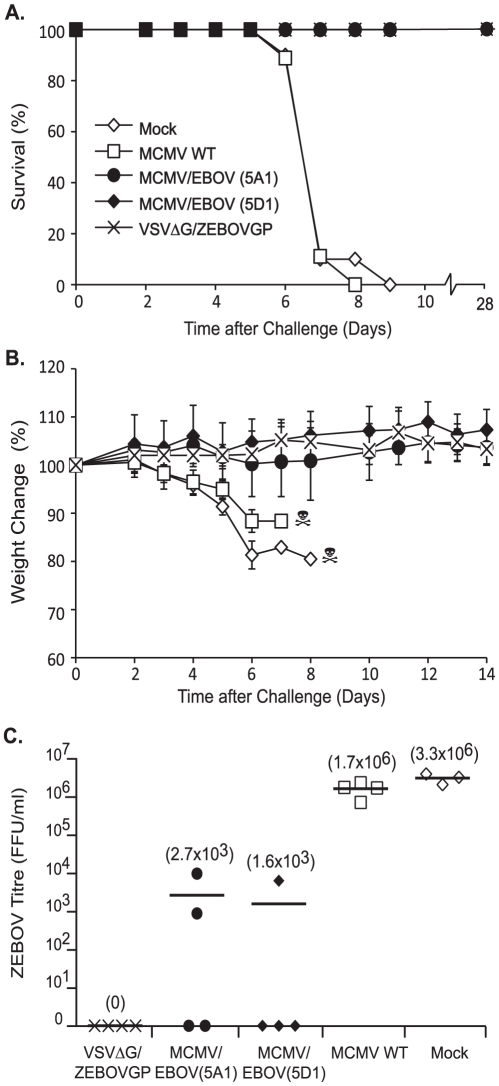
Protective efficacy of MCMV/ZEBOV-NP_CTL_. Groups of C57BL/6 mice (n = 14) were vaccinated by i.p. administration of 5×10^5^ pfu of either MCMV/ZEBOV-NP_CTL_ (clones 5A1 or 5D1), MCMV WT, or diluent (Mock), followed by an identical boost at week 4. An additional group received VSVΔG/ZEBOVGP as a positive control for vaccine efficacy. After 10 weeks (6 weeks after the boost), mice were challenged with 10^3^ LD_50_ ma-ZEBOV (i.p.). Data represent (a) Percent survival. (b) Body weight change over time post-challenge (error bars show the s.d.). (c) Viremia levels in 3–4 mice harvested at time of peak viremia (day 4) (mean viremia levels for each group are shown in parentheses). For body weight, groups were weighed daily until 14 days post-EBOV challenge, or until all animals in a group had succumb to ZEBOV disease. MCMV/ZEBOV-NP_CTL_ vaccination had a significant impact on survival from ma-ZEBOV challenge compared to MCMV WT controls (p<0.0001) using a log-rank test. Analysis of ma-ZEBOV viremia shows a comparable level in control of viremia between 5A1 and 5D1 MCMV/ZEBOV-NP_CTL_ vaccinated groups, compared to MCMV WT controls (p<0.0001).

After 10 weeks (6 weeks following the boost), the remaining mice (n = 14/group) were challenged i.p. with 10^3^ LD_50_ of ma-ZEBOV. An additional group (n = 14) that had received VSVΔG/ZEBOVGP (5×10^5^ pfu; i.p.), which confers high levels of protection against ma-ZEBOV, was included as a control for vaccine protection [24]. ZEBOV disease was then monitored on the basis of survival, morbidity based on clinical signs (ruffled fur, hunched posture, paralysis and weight loss) and viremia. Mock and MCMV WT vaccinated controls exhibited ZEBOV disease and significant morbidity (Figure 4b) with 90% of mice succumbing between days 5–7 post-challenge (Figure 4a). In contrast, MCMV/ZEBOV-NP_CTL_ vaccinated mice showed no evidence of ZEBOV disease, with 100% survival and no signs of morbidity (Figures 4a and 4b). As a quantitative analysis of vaccine efficacy, viremia at day 4 post-challenge (peak of ZEBOV viremia in the mouse model) was measured in a subset of mice (n = 3–4/group) harvested at this time (Figure 4c). MCMV/ZEBOV-NP_CTL_ vaccination resulted in a significant level of control of ZEBOV replication. Specifically, 5 of 8 mice showed no detectable levels of viremia; the remaining 3 mice showed a 2.8-log reduction in viremia compared to WT MCMV vaccinated controls. Given the expression of a single CTL epitope from NP it was highly unlikely that anti-ZEBOV antibodies (either neutralizing or total) would be induced by vaccination. However, it was possible that a low-level of ZEBOV replication in vaccinated animals would result in induction of anti-ZEBOV antibodies. To investigate the possibility that neutralizing antibodies induced by the challenge virus were positively impacting protection, we waited a sufficient period of time (28 days) for any antibodies induced by challenge to have risen to detectable levels. At 28 days post-challenge ma-ZEBOV neutralizing activity in sera from a randomly selected subset (n = 6) of protected MCMV/ZEBOV-NP_CTL_ mice was measured. VSVΔG/ZEBOVGP control mice had low, but detectable levels of neutralizing activity following challenge as previously observed [24]. In contrast, neutralizing activity was not detected in any convalescent serum from MCMV/ZEBOV-NP_CTL_ vaccinated mice demonstrating that the role of neutralizing antibodies in mediating protection in these mice was minimal (Table S1).

Vaccination of either NHPs [22] or mice [24] with VSVΔG/ZEBOVGP is known to induce a total IgG anti-ZEBOV response (presumably directed against ZEBOV GP). Although VSVΔG/ZEBOVGP confers a level of protection that results in the complete lack of detectable ZEBOV viremia [22], the anti-ZEBOV antibody response induced by VSVΔG/ZEBOVGP is subsequently boosted by ZEBOV challenge. An antibody-capture ELISA (using VLPs comprised of VP40, NP and GP as a source of antigen) [53] was used to measure levels of total anti-ZEBOV IgG antibodies in MCMV/ZEBOV-NP_CTL_ vaccinated mice, both prior to challenge and at day 28 post-challenge (Table S2). Consistent with expression of the single NP CTL epitope, anti-ZEBOV antibodies were not detected, or present sporadically at only low levels (in one of three mice tested) in MCMV/ZEBOV-NP_CTL_ vaccinated mice prior to ma-ZEBOV challenge. In contrast, convalescent sera from protected mice in this treatment group had high levels of total IgG directed against ZEBOV (at 28 days post-challenge). VSVΔG/ZEBOVGP-vaccinated mice responded as previously described with anti-ZEBOV IgG antibodies being induced by vaccination, which were then boosted by ma-ZEBOV challenge (Table S2). Whether the anti-ZEBOV antibody response in either of these treatment groups is induced by active ZEBOV replication or represents exposure to the initial antigen bolus received at the time of challenge is unclear. The relatively greater anti-ZEBOV IgG response observed in MCMV/ZEBOV-NP_CTL_ compared to VSVΔG/ZEBOVGP vaccinated mice following challenge may indicate an higher level of ongoing ZEBOV replication in the MCMV/ZEBOV-NP_CTL_ vaccinated mice following challenge. The absence of neutralizing antibodies and barely detectable and sporadic levels of total IgG against ZEBOV in MCMV/ZEBOV-NP_CTL_ vaccinated mice prior to ma-ZEBOV challenge suggests that NP-specific CTL, and not antibodies, are playing a greater role in protection. These results do not exclude other mechanisms being involved in protection, such as neutralizing or non-neutralizing antibodies below the level of detection in our assay, as well as non-specific innate responses induced by the CMV vector itself. Although the inability of WT MCMV to afford any level of protection would presumably suggest minor involvement of non-adaptive, innate responses. *De novo* ZEBOV-specific CTL responses directed against ZEBOV-encoded antigens other than NP induced by the challenge virus can also not be excluded.

Great apes are an important source of EBOV transmission to humans [7], [8], [9], [12], [13]. Vaccination campaigns for rabies in European and US wildlife [60] have shown the effectiveness of targeting animal species involved in transmission. Vaccination of great apes to interrupt EBOV transmission may therefore be an effective strategy to decrease human EBOV outbreaks. In the current study, we demonstrate that a prototype CMV-based vaccine expressing a single CTL NP epitope can induce a considerable level of protection against ZEBOV. The present study therefore establishes a ‘proof-of-concept’ for a CMV-based EBOV vaccine in the C57BL/6 mouse challenge model prior to moving forward with more complex CMV-based vectors (species-specific) expressing full-length EBOV proteins in more robust NHP challenge models. Given the outbred nature of primates with their expression of a diverse repertoire of MHC I alleles, a final CMV based vaccine will assuredly need to encode single or perhaps multiple full-length EBOV proteins.

In addition to being highly immunogenic, CMV has evolved a remarkable ability to spread between individuals, and therefore may be suited for development as a ‘disseminating’ vaccine platform to target geographically inaccessible wild animal populations like great apes at relatively low cost. In this strategy, vaccination of ‘founder vaccine recipients’ would be used to initiate spread of the CMV-based EBOV vaccine through animal populations eliminating the need for immunization of each individual. An important characteristic of CMV that makes this vector ideally suited to development as a ‘disseminating’ vaccine is its remarkable ability to reinfect and establish a persistent infection regardless of host CMV immunity [29], [31], [32], [61]. Recent studies in rhesus macaques show that immunogenicity and capacity to re-infect the CMV immune host is relatively independent of CMV dose, as inoculums as low as 100 pfu of rhesus CMV (RhCMV) were able to reinfect and induce immunity in RhCMV sero-positive animals [62]. As CMV is transmitted through breast milk from mothers to offspring, a CMV-based vaccine could also afford a more permanent solution to the EBOV problem. Although vertical transmission of superantigens encoded by endogenous mammary tumour viruses (MMTV) can cause clonal deletion of reactive T cell subsets [63], vertical transmission of CMV appears to be distinctly different to MMTV, with mature and functional human CMV specific T cells being consistently observed in congenitally infected neonates (and one aborted foetus of 28 weeks gestation) [64], [65].

There are clearly some potential risks associated with use of such a ‘disseminating’ vaccine approach targeting wildlife populations. However, in addition to the impact of EBOV on human health, EBOV is regarded as a major threat to survival of great apes [11]. With potential for achieving high levels of coverage in inaccessible and environmentally harsh regions, a ‘disseminating’ CMV-based EBOV approach may not only benefit humans, but may also positively impact survival of these great ape species in the wild. Concerns regarding the possible environmental impact of releasing a recombinant vaccine vector that can spread within the target population, especially a population that is endangered, cannot be overstated. However a number of additional characteristics of this vaccine approach help to allay these concerns. First, CMV has been shown to be ubiquitous within its respective host population in all NHP species studied [66]–[69]. Therefore, a CMV-based vaccine for use in great ape species represents infection with a benign virus (either gorilla or chimpanzee CMV) that is already present within the population, differing only by expression of EBOV antigens. Second, CMVs are believed to be highly host-specific with each mammalian host studied carrying its own species-specific CMV [70]. This host-specificity is recapitulated to a certain level *in vitro*, with only CMVs from closely related species being able to replicate in cells from other closely related species. For example, human CMV (HCMV) was able to replicate, but at a 10-fold lower level, in primary chimpanzee compared to human fibroblasts [71]. However, HCMV is unable to replicate in mouse fibroblasts [72], and MCMV is reciprocally unable to replicate or is severely compromised for replication in human fibroblasts [72], [73]. For rodent CMVs, the block in cross-species infection has been shown to be due to an inability to control apoptosis following infection of the cross-species cell type [73]. The responsible mechanism for species restriction in primate CMVs is not known.

CMV is ubiquitous in all NHP species studied (baboons, drill monkeys and rhesus macaques) [66]–[68]. A chimpanzee CMV strain (CCMV, *Panine herpesvirus 2*) has been isolated from a chimpanzee in captivity and the genome fully sequenced. The CCMV genome was largely co-linear with that of HCMV, but with a moderate level of divergence [74]. Leendertz *et al* [75] have subsequently detected multiple additional CMV species in samples from captive and wild gorillas (Western Lowland), chimpanzees (West and East African), as well as orangutans using a degenerate PCR-based assay targeting conserved genes. Together with HCMV, as well as the earlier isolated CCMV and old world and new world monkey CMVs, Leendertz *et al* [75] separated primate CMVs into six major clades on the basis of partial sequence of a conserved essential protein, gB. HCMV strains localized within their own clade (93–95% amino acid identity), whilst gorilla and chimpanzee CMVs were contained within two clades [CG1 clade: CCMV, *Pan troglodytes* CMV genogroup 1, and *Gorilla gorilla* CMV genogroup 1 (76–77% amino acid identity with HCMV gB); and CG2 clade: *Pan troglodytes* CMV genogroup 2, and *Gorilla gorilla* CMV genogroup 2 (81–82% amino acid identity with HCMV gB) [75]. Consistent with earlier studies, most of the primate CMVs were found only in their respective host species with which they had co-evolved. However, the presence of chimpanzee and gorilla CMVs together within the same clade raises the possibility of horizontal bi-directional transmission of CMV between chimpanzees and gorillas. CMV detection was based solely on the presence of subgenomic fragments of viral DNA within tissue samples by PCR and not isolation of infectious virus. However, any possibility for transmission between closely related primate species clearly indicates a need for empirical studies to confirm the high species-specificity of CMV between closely related primate species.

In summary, using the mouse ma-ZEBOV challenge model, we have established ‘proof-of-concept’ for CMV as a potential ‘disseminating’ vaccine for EBOV. Future and ongoing studies are focused on the design of CMV vectors expressing full-length EBOV proteins, as well as assessment of efficacy in the NHP model using RhCMV-based vectors. The high immunogenicity, combined with the ability of CMV to spread regardless of prior CMV immunity and host-specificity, make ‘disseminating’ CMV-based vaccines a novel vaccine platform that may be ideal for targeting EBOV, as well as other pathogens, in animal populations that are inaccessible due to geography or vaccination cost.

## Supporting Information

Figure S1 Genomic characterization of MCMV/ZEBOV-NP_CTL_. BAC DNA from two independent clones of MCMV/ZEBOV-NP_CTL_ (5A1 and 5D1) were digested with EcoRI followed by electrophoresis. The comparable digest pattern between MCMV/ZEBOV-NP_CTL_ BAC clones and the MCMV WT BAC shows the lack of any gross genomic rearrangement.
(EPS)

Figure S2 Multi-step growth analysis of MCMV/ZEBOV-NP_CTL_. MEFs were infected at a MOI of 0.1 with either 5A1, 5D1 or WT MCMV. Supernatant was collected at days indicated post-infection and titered by standard plaque assay. The assay was performed in triplicate and standard deviation is shown.
(EPS)

Table S1 (DOC)

Table S2 (DOC)
