# Supplementary material for: A Replicating Cytomegalovirus-Based Vaccine Encoding a Single Ebola Virus Nucleoprotein CTL Epitope Confers Protection against Ebola Virus
Source: PLoS Negl Trop Dis. 2011 Aug 9;5(8):e1275. doi: 10.1371/journal.pntd.0001275 (PMC3153429; doi:10.1371/journal.pntd.0001275)
Supplement: Table S1 — (DOC) [file pntd.0001275.s003.doc]

| **Vaccination** | **Neutralization Titre** |
| --- | --- |
| Mab#226 (positive control)[^1^](#_ENREF_1) | >1280 |
| VSVΔG/ZEBOVGP (Mouse 1) (control) | >40 |
| VSVΔG/ZEBOVGP (Mouse 2) (control) | >80 |
| VSVΔG/ZEBOVGP (Mouse 3) (control) | >40 |
| MCMV/ZEBOV-NP_CTL_ 5A1 (Mouse 1) | Neg |
| 5A1 (Mouse 2) | Neg |
| 5A1 (Mouse 3) | Neg |
| MCMV/ZEBOV-NP_CTL_ 5D1 (Mouse 1) | Neg |
| 5D1 (Mouse 2) | Neg |
| 5D1 (Mouse 3) | Neg |

**Supplementary Table 1.** Neutralizing antibody titre in sera from mice at 28 days following ma-ZEBOV challenge. Sera was collected from vaccinated mice and analyzed for ability to neutralize ZEBOV infection in an *in vitro* neutralization assay[^2^](#_ENREF_2) as detailed in Materials and Methods. Value shown is the sera dilution that results in a 50% reduction in EGFP-positive cells following infection of Vero cells with ZEBOV-EGFP. Dilution (2-fold series) of sera was started at 1/40. Mab#226 is a neutralizing mouse monoclonal antibody made against ZEBOV GP^1^.

1. Takada, A*, et al.* (2003) Identification of protective epitopes on Ebola virus glycoprotein at the single amino acid level by using recombinant vesicular stomatitis viruses. J Virol 77, 1069-1074.

2. Richardson, J.S*, et al.* (2009) Enhanced protection against Ebola virus mediated by an improved adenovirus-based vaccine. *PLoS One* 4, e5308.
