# Supplementary material for: A Replicating Cytomegalovirus-Based Vaccine Encoding a Single Ebola Virus Nucleoprotein CTL Epitope Confers Protection against Ebola Virus
Source: PLoS Negl Trop Dis. 2011 Aug 9;5(8):e1275. doi: 10.1371/journal.pntd.0001275 (PMC3153429; doi:10.1371/journal.pntd.0001275)
Supplement: Table S2 — (DOC) [file pntd.0001275.s004.doc]

| **Vaccination** | **Pre-challenge** | **Post-challenge** |
| --- | --- | --- |
| *Mab#226 (positive control)*[*^1^*](#_ENREF_1) | 6400 | |
| VSVΔG/ZEBOVGP (Mouse 1) (control) | 1600 | 6400 |
| VSVΔG/ZEBOVGP (Mouse 2) (control) | 1600 | 1600 |
| VSVΔG/ZEBOVGP (Mouse 3) (control) | 1600 | 25600 |
| MCMV/ZEBOV-NP_CTL_ 5A1 (Mouse 1) | Neg | 25600 |
| 5A1 (Mouse 2) | Neg | 25600 |
| 5A1 (Mouse 3) | Neg | 6400 |
| MCMV/ZEBOV-NP_CTL_ 5D1 (Mouse 1) | Neg | 6400 |
| 5D1 (Mouse 2) | Neg | 6400 |
| 5D1 (Mouse 3) | 400 | 6400 |

**Supplementary Table 2.** Anti-ZEBOV VLP IgG antibody titre in mouse sera pre- and post- ma-ZEBOV challenge. Sera was collected from vaccinated mice and analyzed for total IgG anti-ZEBOV antibodies using VLPs[^2^](#_ENREF_2) (VP40/NP/GP) as a source of antigen (detailed in Material and Methods). Values shown are the maximal dilution of sera evaluated as positive from a 4-fold dilution series starting at 1/100. Sera were deemed positive if the signal was greater than the mean of Mock mouse sera plus four standard deviations. Mab#226 is a neutralizing mouse monoclonal antibody made against ZEBOV GP^1^.

1. Takada, A*, et al.* (2003) Identification of protective epitopes on Ebola virus glycoprotein at the single amino acid level by using recombinant vesicular stomatitis viruses. J Virol 77: 1069-1074.

2. Wahl-Jensen, V*, et al.* (2005) Role of Ebola virus secreted glycoproteins and virus-like particles in activation of human macrophages. J Virol 79: 2413-2419.
